# Low-grade inflammation from prenatal period to age 6–8 years in a Vitamin D trial

**DOI:** 10.1038/s41390-024-03019-4

**Published:** 2024-01-15

**Authors:** Helena H. Hauta-alus, Jenni Rosendahl, Elisa M. Holmlund-Suila, Saara M. Valkama, Maria Enlund-Cerullo, Markku Nurhonen, Eero Kajantie, Outi Mäkitie, Sture Andersson

**Affiliations:** 1 Children’s Hospital, Pediatric Research Center, University of Helsinki and Helsinki University Hospital, Helsinki, Finland; 2 Research Program for Clinical and Molecular Metabolism (CAMM), Faculty of Medicine, University of Helsinki, Helsinki, Finland; 3 Population Health unit, National Institute for Health and Welfare (THL), Helsinki, Finland; 4 PEDEGO Research Unit, MRC Oulu, Oulu University Hospital and University of Oulu, Oulu, Finland; 5 Folkhälsan Institute of Genetics, Helsinki, Finland; 6 Department of Clinical and Molecular Medicine, Norwegian University of Science and Technology, Trondheim, Norway; 7 Department of Molecular Medicine and Surgery, Karolinska Institutet, and Clinical Genetics, Karolinska University Laboratory, Karolinska University Hospital, SE-17176 Stockholm, Sweden

## Abstract

**Background:**

Low-grade systemic inflammation measured as high sensitivity C-reactive protein (hs-CRP) has been associated with non-communicable disease risk. We assessed whether prenatal inflammation and early-childhood vitamin D are associated with inflammation until age 6–8.

**Methods:**

We analyzed blood hs-CRP and 25-hydroxy vitamin D [25(OH)D] in pregnancy, at birth from umbilical cord blood (UCB), from offspring at ages 1, 2, and 6–8 years in the Vitamin D Intervention in Infants (VIDI) study. VIDI was a randomized-controlled trial of vitamin D supplementation of 10 μg/day or 30 μg/day from age 2 weeks until 2 years in 975 infants recruited in 2013–14, with follow-up at age 6–8 in 2019–21 (*n* = 283).

**Results:**

Pregnancy hs-CRP was associated with UCB hs-CRP (*r* = 0.18, *p* < 0.001) but not independently with childhood hs-CRP (Estimate [95% CI] 0.04 [<−0.00, 0.09]). Higher UCB hs-CRP was associated independently with higher hs-CRP until 6–8 years (0.20 [0.12, 0.29]). Infant vitamin D dose had no effect on longitudinal hs-CRP (6–8 years, 0.11 [−0.04, 0.25]). Childhood 25(OH)D were associated positively with hs-CRP until age 6–8 (0.01 [>0.00, 0.01]).

**Conclusion:**

Our results indicate that in children, inflammation, assessed by hs-CRP, persists from birth until 6–8 years. We observed positive associations between 25(OH)D and hs-CRP in vitamin D-sufficient children.

**Impact:**

- High sensitivity C-reactive protein (hs-CRP) concentrations tract from birth to age 8 years
- Our novel finding suggests a long-lasting pro-inflammatory phenotype in the child
- Higher vitamin D concentration - but not dose – is associated with higher childhood hs-CRP
- Chronic disease risk related to inflammation may in part originate from the prenatal period or early childhood
- Further studies are needed to investigate the effects of inflammation on long-term clinical health outcomes

## Introduction

C-reactive protein (CRP), an acute-phase protein produced in the liver, shows a rapid rise in concentration in response to acute infection, inflammation or injury. With quantitative high-sensitive assays, low concentrations of CRP can also be detectable in apparently healthy individuals. This high-sensitivity CRP (hs-CRP) frequently serves as a marker of low-grade systemic inflammation, which in turn is associated with a wide range of chronic conditions such as obesity, metabolic syndrome, cardiovascular disease and other chronic inflammatory states.^1–4^ Some cardiometabolic risk factors related to inflammation are already evident in childhood.^5^

Inflammation in pregnancy has been associated with neonatal complications such as fetal growth restriction and low birth weight, but its effect on long-term offspring health is unclear.^6,7^ Furthermore, very little is known about the transition of systemic inflammation from mother to child. One hypothesis is that maternal inflammation influences the child’s risk for systemic inflammation, and recent data indicate a correlation between hs-CRP levels in pregnant mothers and in their children.^8,9^

Observational findings have suggested vitamin D as reducing low-grade inflammation.^10–12^ Particularly severe vitamin D deficiency (25-hydroxyvitamin D [25(OH)D] <25 nmol/L) leads to increased hs-CRP, but such an association may become attenuated at higher concentrations of 25(OH)D (>50 nmol/L),^13^ and a report also exists of U-shaped associations.^14^ We have previously reported a correlation between higher 25(OH)D and higher inflammatory markers in cord blood of healthy newborns, with the majority having 25(OH)D above 50 nmol/L.^15^ In the present study, infants participated in a randomized-controlled Vitamin D Intervention in Infants (VIDI) study in which they received daily vitamin D supplementation of 10 µg (400 IU) or 30 µg (1200 IU) for the first two years of life without any effect on infection episodes.^16^

The aim of the present study was to further investigate in a longitudinal study setting, extending from pregnancy until age 6–8 years, whether prenatal and neonatal hs-CRP and early childhood vitamin D are associated with these children’s hs-CRP concentrations. In addition, we describe the determinants of hs-CRP concentration in pregnancy, at birth, and in early childhood.

## Methods

### Study subjects

**Fig. 1:**
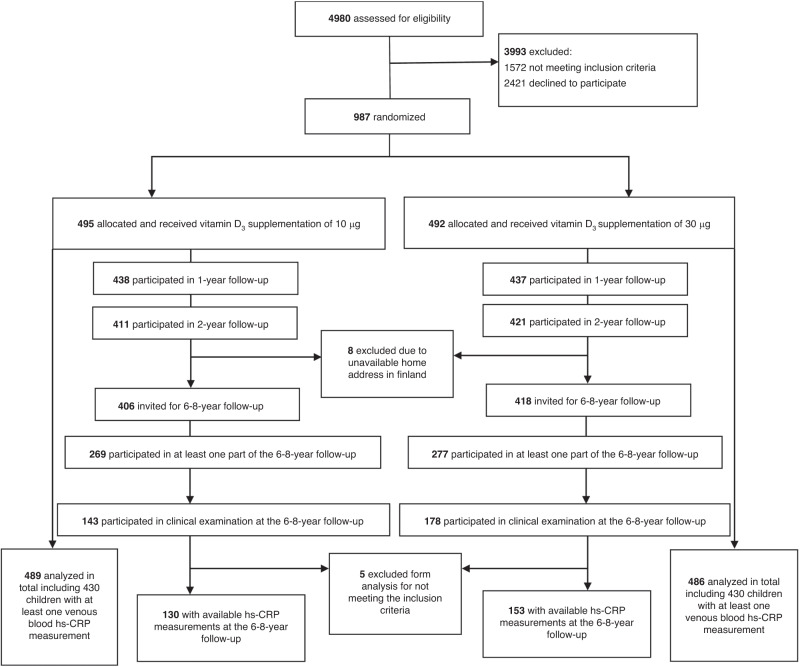
Flow chart of subject recruitment and participation in the study. Number of data available are shown in boxes.

We performed a longitudinal study using data collected in the VIDI study, a randomized controlled trial of daily 10 µg (400 IU) (referred as group 10) or 30 µg (1200 IU) (referred as group 30) vitamin D_3_ supplementation administered to healthy infants from age 2 weeks to 2 years. This protocol has appeared earlier.^16,17^ The VIDI study included 987 healthy infants recruited during 2013–2014 at Maternity Hospital in Helsinki, Finland (Fig. 1). Mothers were of northern European ethnicity, without any regular medication, and with a singleton pregnancy. Infants were born full-term at a birth weight appropriate for gestational age. Exclusion criteria for the infants were intravenous glucose, antibiotic treatment, seizures, nasal continuous positive airway pressure treatment for more than one day, phototherapy for more than three days or nasogastric tube feeding for more than one day. During the intervention, study visits took place at the ages of 6 months, and 1 and 2 years, and were completed in June 2016.

We began the VIDI follow-up study (VIDI2) in 2019 when participants were turning 6 years of age. Because of the COVID-19 pandemic, we were able to complete our study when the children were 6–8 years of age (mean age 7) between November 2019 and December 2021. For VIDI2 study, we invited all those 824 participants who completed VIDI study’s 2-year visit and had a valid home address in Finland at time of recruitment (Flow chart of subject participation, Fig. 1). Of those invited, 546 (66%) participated in at least one part of the study, and of those invited 321 (39%) participated in clinical examination at the Clinical Trial Unit of the new Children’s Hospital, Helsinki; 283 had data on hs-CRP available. In the current analyses, we excluded 12 subjects, of whom 5 attended the VIDI2 clinical examination, for not meeting the inclusion criteria, resulting in a total of 975 subjects. The number of subjects varies among the analyses due to partial missing data, and are reported in the tables.

All parents gave their written informed consent, and for VIDI2, also the children provided informed assent. The Research Ethics Committee of the Hospital District of Helsinki and Uusimaa granted ethical approval (107/13/03/03/2012; HUS/1541/2019). The study was conducted in accordance with the Declaration of Helsinki. Study protocols are registered at ClinicalTrials.gov (NCT01723852; NCT04302987).

### Maternal and child data

Maternal, parental and child characteristics originated in medical records, and in addition, subjects completed self-administered structured questionnaires at recruitment and during study visits. Mother’s pre-pregnancy body mass index (BMI, kg/m²) was calculated from weight and height. Maternal dietary patterns were based on a 22-item semi-quantitative food frequency questionnaire (FFQ) representing eating habits at the end of pregnancy. Of the five different dietary patterns identified,^18^ we chose based on literature a “health-conscious dietary pattern” for analyses, which was dominated by high consumption of fresh and cooked vegetables, fruits/berries, fish, and seeds/beans. Maternal education level was categorized into “lower” and “higher” education (lower = lower or upper secondary or post-secondary non-tertiary education or less than a bachelor’s degree, higher = first or second stage of tertiary education or at least a bachelor’s degree).

At the study visits, study personnel evaluated the child for any symptoms of current infection. Children were measured for length or height and weight, and length or height, and length- or height-adjusted weight we expressed as standard deviation scores (SDS) using age- and sex-specific Finnish references.^19,20^ The mothers’ and children’s allergies were assessed using structured allergy questionnaires based on the modified International Study of Asthma and Allergies in Childhood (ISAAC) questionnaire.^21^

### Laboratory analyses

Samples were obtained at five time points: during pregnancy from maternal serum, at birth from umbilical cord blood (UCB), and at ages 1, 2, and 6–8 years from the child’s serum. Serum samples from the pregnant mothers were collected on average at 11 weeks of gestation during routine follow-up visits at prenatal clinics and were stored in the Finnish Maternity Cohort serum bank maintained by the National Institute for Health and Welfare. Hs-CRP concentration we determined with an enzyme immunoassay (IBL international, and Demeditec CRP high-sensitive ELISA) according to manufacturer’s instructions. The assay detection limit was 0.02 µg/ml. Pregnancy hs-CRP showed 12 values above 120 µg/ml after maximum amount of dilution (1:10,000) and were determined to exactly 120 µg/ml. Similarly, for childhood hs-CRP at 1 year 6 values were above 100 µg/ml, at 2 years 4 values were above 50 µg/ml and 5 values above 60 µg/ml after maximum amount of dilutions. These values were estimated to exact values of 100 µg/ml, 60 µg/ml, and 50 µg/ml, respectively, based on absorbance value compared with standard curve. Maximum number of dilution was set to preserve reliability in high-sensitivity kits. Concentration of 25(OH)D we analyzed with a fully automated immunoassay (IDS-iSYS, Immunodiagnostic System Ltd., Bolton, UK). Details of the maternal and infant 25(OH)D measurements have been provided earlier.^16,22^ At 6–8 years of age, intra-assay variation for 25(OH)D via the IDS-iSYS method was <3%, and applying seven standardized samples revealed the method to show a <5% positive bias (Pharmatest, Turku, Finland).

### Statistical analyses

Childhood hs-CRP concentrations served as secondary outcome measures. Normality of continuous variables was determined by visual inspection. Hs-CRP concentration was log-transformed prior to analysis. For group comparisons, we used the Independent samples T-test, Pearson Chi-Square, Kruskal–Wallis or Mann–Whitney U-test according to distribution of the variable. Factors associated with hs-CRP concentration were analyzed with univariate linear regression analysis at each of the five time-points separately. We categorized serum 25(OH)D into groups of <75 nmol/L, 75–125 nmol/L, and >125 nmol/L, because the number of subjects with 25(OH)D < 50 nmol/L was limited.

To investigate the tracking of prenatal hs-CRP (pregnancy hs-CRP; UCB hs-CRP) and the effect on child’s hs-CRP of early childhood vitamin D intervention during 6–8 years’ follow-up we applied a linear mixed effects model with R package lme4. This allowed us to test repeated measures and to include subjects with partial missing data. Due to skewness of log-transformed hs-CRP values, models were run with a bootstrapping procedure with the R package lmeresampler (number of samples = 2000). We chose covariates based on consistent pairwise correlations with dependent hs-CRP and on the literature. Results are shown separately for unadjusted models and models adjusted for parity, pre-pregnancy BMI, and smoking, maternal education, mode of delivery, antibiotic use during delivery, sex, number of siblings at sample collection, acute infection at sample collection, length-adjusted weight SDS at sample collection, and vitamin D intervention group (in analyses of prenatal hs-CRP). No interaction with time was detectable (*p* > 0.25).

All analyses utilized SPSS software (IBM SPSS Statistics for Windows, version 28) and R statistical environment (RStudio version 2022.07.2 Build 576).

## Results

**Fig. 2:**
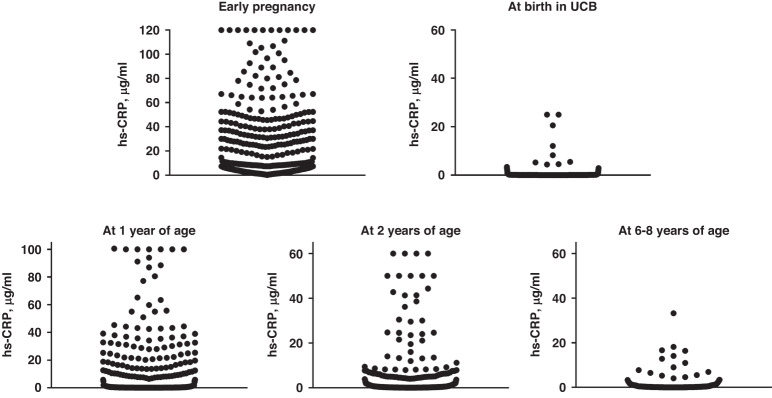
High-sensitivity C-reactive protein (hs-CRP) concentrations at five different time points. Variation of hs-CRP concentrations are shown in mother-offspring pairs at early pregnancy (*n* = 807), at birth from umbilical cord blood (UCB) (*n* = 956) and at offspring ages of 1 (*n* = 748), 2 (*n* = 789), and 6–8 years (*n* = 283).

The study cohort included a total of 975 children and their mothers whose main baseline characteristics are presented in Table 1 separately for subjects with and without measured hs-CRP at age 6-8 years. The majority of the mothers were primipara, with a high educational level and vaginal delivery. Of all 891 mothers 655 (74%) had normal pre-pregnancy BMI. Mothers of children with their hs-CRP at VIDI2 follow-up study measured at age 6–8 years were older, smoked less, had more allergic diseases and a higher education, more often received antibiotic treatment during labor, and had higher maternal 25(OH)D than did those mothers whose children had no hs-CRP measurement at age 6–8 (Table 1). Figure 2 demonstrates the variation in hs-CRP at all time points. Hs-CRP in pregnancy and in UCB correlated positively (Spearman r = 0.18, *p* < 0.001). Median (IQR) values for hs-CRP was 0.20 (0.78) in 748 children at 1 year, was 0.29 (0.75) in 789 at 2 years, and was 0.23 (0.59) in 283 at 6–8 years.

**Table 1:** Maternal and infant characteristics comparing subjects with and without measured hs-CRP levels at 6–8 years.

Mother	Subjects without measured hs-CRP at 6–8 years (*n* = 692)	Subjects with measured hs-CRP at 6–8 years (*n* = 283)	*p* value^a^
Age, years^b^	31.2 (4.6)	32.1 (4.1)	**0.004**
Parity, primipara	378/561 (62)	183/283 (65)	0.46
Pre-pregnancy BMI, kg/m^2^	23.2 (3.8) [*n* = 608]	23.3 (3.6)	0.65
Pre-pregnancy smoking, yes	101/597 (17)	29/281 (10)	**0.010**
Allergic disease	248/555 (45)	152/282 (54)	**0.012**
Education, higher	431/600 (72)	226/283 (80)	**0.011**
Gestational diabetes	74/692 (11)	37/283 (13)	0.29
Antibiotic treatment during labor	168/692 (24)	88/283 (31)	**0.028**
Mode of delivery, vaginal	650/692 (94)	261/283 (92)	0.33
Gestational age at blood sampling, weeks, md (IQR)	11.1 (1.6) [*n* = 568]	10.9 (1.6) [*n* = 240]	**0.033**
Pregnancy hs-CRP, µg/ml, md (IQR)	4.68 (7.42) [*n* = 569]	4.74 (6.71) [*n* = 238]	0.79
Pregnancy hs-CRP ≥ 10 µg/ml	136/569 (24)	47/240 (20)	0.18
Pregnancy 25(OH)D, nmol/l	80.6 (19.1) [*n* = 569]	84.7 (22.5) [*n* = 240]	**0.008**
Infant			
Female sex	346/692 (50)	139/283 (49)	0.80
Gestational age, weeks	40.2 (1.1)	40.2 (1.1)	0.82
UCB 25(OH)D, nmol/l	79.4 (23.0) [*n* = 680]	86.7 (31.4) [*n* = 275]	**<0.001**
UCB hs-CRP, µg/ml, md (IQR)	0.06 (0.05) [*n* = 680]	0.06 (0.05) [*n* = 276]	0.40
UCB hs-CRP ≥ 10 µg/ml	4/956 (<1)	0/276 (0)	
Birth weight, kg	3.54 (0.40)	3.53 (0.39)	0.57
Length-adjusted birth weight, SDS	0.11 (0.95)	0.05 (0.94)	0.32
Breastfed > 6 months	448/572 (78)	226/282 (80)	0.54

*SD* standard deviation, *md* median, *IQR* interquartile range, *SDS* standard deviation score (based on Finnish normative data on birth size), *BMI* body mass index, *hs-CRP* high-sensitive C-reactive protein, *25(OH)D* 25-hydroxy vitamin D, *UCB* umbilical cord blood.

Values are means (SD) or *n*/*N* (%), with number of subjects 692 or 283 unless otherwise indicated.

*p* values below 0.05 are shown in bold.

^a^Statistical comparison by independent samples T-test and Mann–Whitney U-test, and Pearson Chi-Square when adequate number of subjects.

^b^Age at baseline.

Most of the 6–8-year-olds in each intervention group had a sufficient 25(OH)D concentration (≥50 nmol/L) (all: 271/287, 94%; group 10: 125/131, 95%; group 30: 146/156, 94%, *p* = 0.50). None had 25(OH)D below 30 nmol/L. Mean (SD) value of 25(OH)D among the 287 at 6–8 years of age was 71.3 (15.7) nmol/L without any differences in the intervention groups (*p* = 0.08). At age 1, infants in group 30 had higher prevalence of cow’s milk allergy than did those in group 10,^23^ but this difference was no longer evident at ages 2 and 6–8 years (data not shown).

### Factors associated with hs-CRP levels at various time points in pregnancy, birth and childhood

Univariate and pairwise associations of sociodemographic and physical factors with hs-CRP at each time-point are presented in Table 2. Factors associating with higher maternal hs-CRP were higher pre-pregnancy BMI, gestational diabetes, smoking, antibiotic treatment during labor, cesarean section, and higher 25(OH)D concentration. Median pregnancy hs-CRP concentration was higher in mothers with pre-pregnancy BMI > 25 kg/m² (7.9 [IQR 29.0]) than in those with BMI ≤ 25 kg/m² (3.9 [IQR 5.7] (*p* < 0.001). Factors associated with lower maternal hs-CRP were higher adherence to a health-conscious dietary pattern, higher education, and multiparity (Table 2).

**Table 2:** Univariate and pairwise associations between possible inflammatory factors and hs-CRP levels in pregnancy, at birth, and at ages 1, 2, and 6–8 years.

Factors associated with pregnancy hs-CRP				
	*n*	B (95% CI)	*Beta*	*p* value
Parity, multipara (*n* = 271) vs primipara (*n* = 475)	746	0.04 (−0.16, 0.24)	0.02	0.69
Pre-pregnancy BMI, kg/m^2^	745	0.12 (0.10, 0.15)	0.35	**<0.001**
Pre-pregnancy smoking, yes (*n* = 111) vs no (*n* = 621)	732	0.22 (−0.05, 0.49)	0.06	0.10
Maternal allergic disease, yes (*n* = 338) vs no (*n* = 361)^a^	699	0.16 (−0.03, 0.36)	0.06	0.10
Maternal health conscious dietary pattern score	574	−0.21 (−0.32, −0.11)	−0.16	**<0.001**
Maternal education, higher (*n* = 549) vs lower (*n* = 188)	737	−0.29 (−0.51, −0.08)	−0.10	**0.009**
Gestational diabetes, yes (*n* = 89) vs no (*n* = 718)	807	0.38 (0.09, 0.69)	0.09	**0.011**
25(OH)D, nmol/l	807	>0.00 (< −0.00, 0.01)	0.01	0.78
Factors associated with UCB hs-CRP at birth				
Parity, multipara (*n* = 326) vs primipara (*n* = 548)	874	−0.24 (−0.36, −0.12)	−0.13	**<0.001**
Pre-pregnancy BMI, kg/m^2^	873	0.03 (0.02, 0.05)	0.13	**<0.001**
Pre-pregnancy smoking, yes (*n* = 127) vs no (*n* = 733)	860	0.16 (0.01, 0.36)	0.07	**0.034**
Maternal allergic disease, yes (*n* = 391) vs no (*n* = 429)^a^	820	0.02 (−0.10, 0.15)	0.01	0.71
Maternal health conscious dietary pattern score	574	−0.05 (−0.13, 0.02)	−0.06	0.16
Maternal education, higher (*n* = 647) vs lower (*n* = 218)	865	−0.06 (−0.20, 0.08)	−0.03	0.41
Gestational diabetes, yes (*n* = 110) vs no (*n* = 846)	956	0.12 (−0.07, 0.30)	0.04	0.21
Antibiotic treatment during labor, yes (*n* = 253) vs no (*n* = 703)	956	0.44 (0.31, 0.57)	0.21	**<0.001**
Mode of delivery, cesarean section (*n* = 64) vs vaginal (*n* = 892)	956	0.33 (0.10, 0.56)	0.09	**0.005**
Length-adjusted weight at birth, SDS	956	−0.03 (−0.09, 0.03)	−0.03	0.32
25(OH)D, nmol/l	952	>0.00 (0.00, 0.01)	0.08	**0.021**
Factors associated with hs-CRP at age 1 year				
Sex, female (*n* = 377) vs male (*n* = 371)	748	0.30 (−0.01, 0.60)	0.07	0.05
Breastfed, ≤6 months (*n* = 154) vs >6 months (*n* = 580)	734	−0.16 (−0.53, 0.22)	−0.03	0.40
Number of siblings	748	0.23 (0.04, 0.42)	0.09	**0.017**
Daycare attendance, yes (*n* = 32) vs no (*n* = 716)	748	0.81 (0.0, 1.55)	0.08	**0.032**
Parental smoking, yes (*n* = 81) vs no (*n* = 576)	657	0.08 (−0.41, 0.56)	0.01	0.75
Maternal allergic disease, yes (*n* = 341) vs no (*n* = 383)^a^	724	0.16 (−0.14, 0.47)	0.04	0.30
Maternal education, higher (*n* = 555) vs lower (*n* = 184)	739	−0.14 (−0.49, 0.21)	−0.03	0.45
Length-adjusted weight, SDS	747	0.05 (−0.19, 0.20)	0.03	0.50
Cow’s milk allergy, yes (*n* = 27) vs no (*n* = 634)	661	0.27 (−0.55, 1.08)	0.03	0.52
Eczema, yes (*n* = 113) vs no (*n* = 552)	665	0.37 (−0.06, 0.79)	0.07	0.09
Current infection, yes (*n* = 70) vs no (*n* = 678)	748	1.57 (1.07, 2.08)	0.22	**<0.001**
Vitamin D supplementation, group-30 (*n* = 379) vs group-10 (*n* = 369)	748	0.20 (−0.10, 0.50)	0.05	0.19
25OHD, nmol/l	739	0.01 (>0.00, 0.02)	0.13	**<0.001**
Factors associated with hs-CRP at age 2 years				
Sex, female (*n* = 399) vs male (*n* = 390)	789	0.26 (0.03, 0.49)	0.08	**0.028**
Number of siblings	728	−0.05 (−0.21, 0.10)	−0.02	0.52
Daycare attendance, yes (*n* = 470) vs no (*n* = 294)	764	0.55 (0.31, 0.80)	0.16	**<0.001**
Parental smoking, yes (*n* = 133) vs no (*n* = 649)	782	−0.20 (−0.51, 0.12)	−0.04	0.22
Maternal allergic disease, yes (*n* = 380) vs no (*n* = 406)^a^	786	0.03 (−0.21, 0.26)	0.01	0.83
Maternal education, low (*n* = 188) vs high (*n* = 596)	784	0.07 (−0.21, 0.34)	0.02	0.64
Length-adjusted weight, SDS	786	0.13 (0.01, 0.24)	0.07	**0.040**
Cow’s milk allergy, yes (*n* = 32) vs no (*n* = 746)	778	−0.23 (−0.82, 0.36)	−0.03	0.44
Eczema, yes (*n* = 145) vs no (*n* = 635)	780	0.07 (−0.23, 0.37)	0.02	0.66
Current infection, yes (*n* = 223) vs no (*n* = 565)	788	1.14 (0.90, 1.39)	0.31	**<0.001**
Vitamin D supplementation, group-30 (*n* = 395) vs group-10 (*n* = 394)	789	0.06 (−0.17, 0.29)	0.02	0.61
25(OH)D, nmol/l	788	0.01 (>0.00, 0.01)	0.10	**0.004**
Factors associated with hs-CRP at age 6−8 years				
Sex, female (*n* = 139) vs male (*n* = 144)	283	0.61 (0.23, 0.98)	0.19	**0.002**
Number of siblings	283	−0.01 (−0.28, 0.25)	−0.01	0.92
Parental smoking, yes (*n* = 52) vs no (*n* = 231)	283	0.06 (−0.44, 0.55)	0.01	0.82
Maternal allergic disease, yes (*n* = 152) vs no (*n* = 130)^a^	282	0.14 (−0.25, 0.52)	0.04	0.49
Maternal education, low (*n* = 57) vs high (*n* = 226)	283	0.08 (−0.40, 0.56)	0.02	0.74
Length-adjusted weight, SDS	282	0.52 (0.33, 0.71)	0.30	**<0.001**
Cow’s milk allergy, yes (*n* = 15) vs no (266)	281	−0.19 (−1.04, 0.67)	−0.03	0.67
Eczema, yes (*n* = 57) vs no (*n* = 225)	282	>0.00 (−0.48, 0.48)	0.00	0.99
Current infection, yes (*n* = 6) vs no (*n* = 276)	282	1.68 (0.37, 2.99)	0.15	**0.012**
Vitamin D supplementation, group-30 (*n* = 153) vs group-10 (*n* = 130)	283	0.12 (−0.26, 0.51)	0.04	0.53
25(OH)D, nmol/l	279	0.01 (−0.01, 0.02)	0.05	0.41

Univariate linear regression applied with log-transformed hs-CRP. B indicates unstandardized coefficient and *Beta* indicate standardized coefficient.

*p* values below 0.05 are shown in bold.

^a^Maternal allergy based on a positive answer in any questionnaires at ages 1, 2, and 6–8 years.

In these children, at all time points, acute infection was associated with higher concurrent hs-CRP level. At ages 1 and 2 years, factors associating with higher concurrent hs-CRP were higher number of siblings, daycare attendance, higher length-adjusted weight, female sex, and higher 25(OH)D concentration. At 6–8 years of age, female sex and higher height-adjusted weight associated with higher hs-CRP. No linear association existed between 25(OH)D and hs-CRP at age 6–8 (Table 2).

Based on their 25(OH)D concentration at time points 1, 2, and 6–8 years, we divided subjects into three groups (<75, 75–125 and >125 nmol/L). We observed the highest hs-CRP (in log-scale) in those subjects with the highest 25(OH)D at age 1 year (median [IQR] in group <75 nmol/L: 0.17 [0.56], *n* = 151; in group 75–125 nmol/L: 0.19 [0.56], *n* = 457; in group >125 nmol/L: 0.41 [1.65], *n* = 131, respectively; *p* = 0.005), and at age 2 years (in group <75 nmol/L: 0.21 [0.63], *n* = 129; in group 75–125 nmol/L: 0.31 [0.66], *n* = 491; in group >125 nmol/L: 0.31 [1.24], *n* = 168, respectively; *p* = 0.020 [pairwise significance only between <75 nmol/L and >125 nmol/L, *p* = 0.016]). At age 6–8 only one subject had 25(OH)D above 125 nmol/L. A comparison was thus made between the groups with <75 nmol/L and ≥75 nmol/L, with a higher hs-CRP observable in the higher 25(OH)D group (in group <75 nmol/L: 0.19 [0.44], *n* = 187; in group ≥75 nmol/L: 0.26 [0.99], *n* = 92, respectively, *p* = 0.030).

### Tracking of prenatal hs-CRP and childhood vitamin D in a longitudinal setting

Table 3 shows the tracking of prenatal hs-CRP to child hs-CRP until age 2 and 6–8 years with a longitudinal design. Early pregnancy hs-CRP was associated with offspring hs-CRP until 2 and age 6–8 but that association became attenuated after adjustment for parity, maternal pre-pregnancy BMI, and smoking, maternal education, mode of delivery, antibiotic use during delivery, sex, number of siblings at sample collection, acute infection at sample collection, length/height-adjusted weight SDS at sample collection, and vitamin D intervention group. However, UCB hs-CRP was associated with offspring hs-CRP levels until age 2 and age 6–8 also when adjusted. As a sensitivity analysis, we excluded the 12 women with pregnancy hs-CRP values above 120 µg/ml; this, however, left the results significantly unchanged.

**Table 3:** Tracking of low-grade inflammation and infant vitamin D on child’s longitudinal hs-CRP concentration during follow-up periods until 2 and 6–8 years of age.

	Child’s hs-CRP during 2 years of follow-up	
	Unadjusted	Adjusted^c^
Pre-and neonatal inflammation	Estimate (Perc 95% CI)	Estimate (Perc 95% CI)
Pregnancy hs-CRP^a^	0.06 (0.02, 0.11)*	0.03 (−0.01, 0.09)
UCB hs-CRP^b^	0.15 (0.07, 0.25)*	0.23 (0.15, 0.34)*
Infant vitamin D^b^		
Early childhood vitamin D supplementation, group-30 vs group-10	0.13 (−0.03, 0.28)	0.13 (−0.05, 0.28)
25(OH)D concentration at 1 year of age	0.01 (>0.00, 0.01)*	0.01 (>0.00, 0.01)*
25(OH)D concentration during 2 years follow-up^d^	0.01 (>0.00, 0.01)*	0.01 (>0.00, 0.01)*
Pre-and neonatal inflammation	Child’s hs-CRP during 6–8 years of follow-up	
Pregnancy hs-CRP^a^	0.07 (0.03, 0.12)*	0.04 (< −0.00, 0.09)
UCB hs-CRP^b^	0.14 (0.07, 0.23)*	0.20 (0.12, 0.29)*
Infant vitamin D^b^		
Early childhood vitamin D supplementation, group-30 vs group-10	0.12 (−0.04, 0.27)	0.11 (−0.04, 0.25)
25(OH)D concentration at 1 year of age	0.01 (>0.00, 0.01)*	0.01 (>0.00, 0.01)*
25(OH)D concentration during 6–8 years follow-up^d^	0.01 (>0.00, 0.01)*	0.01 (>0.00, 0.01)*

Bootstrapped estimate with percentile (Perc) 95% confidence intervals (95% CI) of mixed effects model applying log-transformed hs-CRP. Children with at least one hs-CRP measurement at 1, 2 or 6–8 years of age: n = 860, and children with hs-CRP at 6–8 years of age: n = 283.

*UCB* umbilical cord blood, *group-10* vitamin D intervention group receiving 10 µg/day the first 2 years of life, *group-30* vitamin D intervention group receiving 30 µg/day the first 2 years of life, *SDS* standard deviation score (based on Finnish normative data of child size), *hs-CRP* high-sensitive C-reactive protein, *25(OH)D* 25-hydroxy vitamin D.

*p < 0.05.

^a^Dependent as repeated measures of hs-CRP at birth from UCB, 1 year, 2 (and 6–8) years of age from venous blood.

^b^Dependent as repeated measures of hs-CRP at 1 year, 2 (and 6–8) years of age from venous blood.

^c^Adjusted for parity, pre-pregnancy BMI and smoking, maternal education, mode of delivery, antibiotic use during delivery, sex, number of siblings at sample collection, acute infection at sample collection, length-adjusted weight SDS at sample collection, and vitamin D intervention group (in analyses of maternal hs-CRP).

^d^Serum 25(OH)D as repeated measures at age 1 year, 2 (and 6–8).

The dose of early childhood vitamin D supplementation showed no association with childhood hs-CRP in a longitudinal setting (Table 3). We observed partial pairwise associations between higher 25(OH)D and higher concurrent hs-CRP (Table 2), and longitudinal associations of higher infant and childhood 25(OH)D with higher hs-CRP until age 2 and until 6–8 years of age (Table 3). Maternal 25(OH)D was not associated with child’s hs-CRP at age 1-, 2- or at 6–8 years (*p* > 0.1 for all).

## Discussion

In this large longitudinal study of pregnant mothers and their offspring, we examined the transmission of systemic inflammation from mother to child by repeated measurements of hs-CRP in early pregnancy, at birth, and at ages 1, 2, and 6–8 years. Independent associations emerged between UCB hs-CRP concentration and childhood hs-CRP up to age 6–8 years. In addition, we confirmed protective factors and risk factors for systemic inflammation in the perinatal period and during childhood. Our results indicate that vitamin D played a role in inflammation, but not through vitamin D supplementation dose but rather by individual 25(OH)D concentration, as higher vitamin D status associated with higher hs-CRP levels in childhood. These findings are important for understanding the mechanisms of systemic inflammation to aid in developing strategies to reduce inflammation-related chronic-disease risk later in life.

We found evidence of tracking of systemic inflammation from mother to child. Our finding is similar to that in the COPSAC cohort in which pregnancy hs-CRP level correlated with offspring hs-CRP at age 6 months.^8^ Despite detecting no independent correlation between early pregnancy and childhood hs-CRP, we did observe an independent association of hs-CRP in UCB at birth with childhood hs-CRP until age 6–8 years. Our early-pregnancy blood sample was from median gestational week 11, while in the COPSAC study samples were from gestational week 24. It is possible that prenatal inflammation does not track into later childhood but only until early infancy. Further, our results imply that other factors might be stronger determinants of offspring hs-CRP after birth than is early-pregnancy hs-CRP. Our pregnancy hs-CRP levels were higher than were our offspring hs-CRP, in line with earlier observations of hs-CRP levels being elevated in healthy pregnant women compared with non-pregnant women.^24,25^

Intergenerational inflammation could be explained in part by modifications in the inflammatory response induced by maternal and child obesity.^26,27^ In line with others, we observed that hs-CRP concentrations were higher at higher adiposity levels, measured as BMI in mothers and length-/height-adjusted weight in children.^1,24,28^ Obesity is a known risk factor for chronic low-grade inflammation: excess nutrients and energy in metabolic cells activate immune cells, triggering inflammatory responses in tissues.^29^ Maternal pre-pregnancy obesity is associated with increased risk of obesity and adverse cardiovascular and metabolic outcomes in the offspring.^30,31^ The mechanism behind these associations is unclear but may involve an increased pro-inflammatory state related to obesity during pregnancy and childhood.^26,29^ For example, the maternal obesity-related inflammatory environment might induce programming of offspring appetite, gene expression, immunity, gut microbiota, and adipocyte function.^32^

A maternal health-conscious dietary pattern was inversely associated with pregnancy hs-CRP concentration. Our finding is consistent with other findings in adults and children.^7,33–37^ This suggests that such practices as increased fruit and vegetable intakes are beneficial in preventing low-grade inflammation with possible intergenerational effects,^38^ although part of the effect might be explained by adiposity.^39^ In line with earlier observations, pre-pregnancy smoking was associated with higher UCB hs-CRP.^40,41^ Often such lifestyle and socioeconomic factors accumulate in same individuals and families making it challenging to reveal independent effects.

Vitamin D exerts its immunomodulatory effects on both the innate and the adaptive immune systems. The active metabolite of vitamin D, 1,25OH_2_D, inhibits the synthesis of interleukin 6, a CRP activator.^42^ Observational studies demonstrate associations between lower vitamin D concentration and increased inflammatory markers^9,13^ but also demonstrate positive and U-shaped associations between 25(OH)D and hs-CRP.^14,43^ We earlier reported a positive cross-sectional correlation between 25(OH)D and hs-CRP in cord blood of healthy newborns recruited in the VIDI cohort.^15^ In the present longitudinal follow-up of that cohort, we observed that the positive correlation between vitamin D concentration and hs-CRP persists in the children until age 6–8 years. The VIDI study included mostly vitamin D-sufficient children, and in fact, 25(OH)D concentrations were exceptionally high in this cohort, with the highest hs-CRP occurring in children with highest 25(OH)D concentration at all time points. This suggests that vitamin D concentration may have inflammatory effects at high levels. As we lacked vitamin D-deficient children, we could not evaluate the effect of low vitamin D status on inflammation. Nonetheless, we found no effect of early childhood vitamin D supplementation dose on hs-CRP concentrations during the first 2 or 6–8 years of life, in accordance with other randomized clinical trials investigating solely vitamin D supplementation.^44–48^

The strength of our study is its longitudinally collected data until age 6–8 years, which provided an opportunity to study relationships involving hs‐CRP levels over time. Our study cohort was large and homogenous, with rigorously collected data on mother-child pairs from early pregnancy to children 6–8 years old. A limitation is that this study was in part observational and does not allow for confirmation of causality. The associations observed were relatively weak and could have arisen by chance or be due to residual confounding. We did, however, adjust analyses for all potential confounders. Another limitation was that for a few high hs-CRP values we were not able determine the accurate concentration but the estimate concentration. Excluding high hs-CRP pregnancy samples from analyses did not, however, change results. Acute infection is probably the most important modifier of hs-CRP concentrations, and we acknowledge that regarding the early pregnancy samples, the possibility of acute infection could not be ruled out. However, at birth and at the child follow-ups, we took possible infections into account; due to the study inclusion criteria, the participating newborns were healthy, and during child follow-up visits, trained study personnel evaluated each child for possible symptoms of infection. Because our participating families were more highly educated than is the general population in Finland, generalizability may be in some doubt. We also lost individuals during follow-up in part due to the pandemic, with only 33% of the children of the original cohort being clinically monitored at age 6–8. These children who participated at age 6–8 differed from the non-participants by a few maternal factors: maternal smoking, for instance, was more common in non-participants than in our participant mothers. It seems unlikely, however, that these factors would have caused bias in our present analysis.

## Conclusions

Persistent association of inflammation in childhood: from birth up to age 6–8 years, suggests a trajectory of a pro-inflammatory phenotype. Furthermore, our findings show consistent association between higher 25(OH)D concentration —but not vitamin D supplementation dose—and higher childhood hs-CRP. Further studies are essential to confirm our findings and to evaluate the possible effects of intergenerational inflammation on long-term offspring health.

## Data Availability

Individual-level data will not be shared publicly, because it is confidential; however, upon reasonable request, data are available from the corresponding author.
